# Effect of Fumed Silica Nanoparticles on Ultraviolet Aging Resistance of Bitumen

**DOI:** 10.3390/nano11020454

**Published:** 2021-02-11

**Authors:** Goshtasp Cheraghian, Michael P. Wistuba

**Affiliations:** Braunschweig Pavement Engineering Centre, Technische Universität Braunschweig, 38106 Braunschweig, Germany; m.wistuba@tu-braunschweig.de

**Keywords:** modified asphalt binder, fumed silica, bitumen aging, nanoparticles

## Abstract

In this study, bitumen modified by fumed silica nanoparticles was characterized through dynamic shear rheometer tests, scanning electron microscopy, and Fourier transform infrared spectroscopy. The fumed silica nanoparticles were used in three different ratios, i.e., 0.1, 0.2 and 0.3 wt.-% of bitumen. Specifically, the modified bitumen characteristics were studied after laboratory aging by analyzing the chemical composition and rheological properties. From the determination of oxidation degree and carbonyl index it was found that the resistance of the modified bitumen to ultraviolet aging was improved with the increasing nanoparticle content. In bitumen modified by fumed silica nanoparticles, the nanoparticles were well dispersed. Moreover, the results illustrated that the bitumen properties were improved, and the improvement effect of 0.1 wt.-% fumed silica nanoparticles was more distinct than the higher concentrations.

## 1. Introduction

Asphalt aging includes thermo oxidative short-term, thermal long-term, and ultraviolet aging. Thermo oxidative aging mostly happens during storage, mixing, and construction, whereas UV aging mostly happens in service. Aging is an inevitable trend which causes brittleness and stiffness in bitumen and considerably decreases the pavement service life [1,2,3,4]. As for thermal aging, bitumen molecules react with oxygen molecules, and hence, bitumen changes its molecular structure. As regards ultraviolet aging, bitumen partially absorbs ultraviolet light originating from sun radiation, and consequently, its molecular structure and chemical bonds change [5]. In most laboratory studies on bitumen aging, the effect of UV light is ignored [6]. Moreover, unlike for thermo oxidative short-term and thermal long-term aging, there is no standard protocol for UV aging [7,8]. UV aging increases the hardness and brittleness of bitumen, which are dominant factors for the resistance of asphalt pavements to cracking [9].

UV radiation includes three wavelength groups:

➢ UV radiation A with a wavelength of 315–400 nanometers which accounts for 8% of total sun UV radiation;
➢ UV radiation B with a wavelength of 280–315 nanometers which accounts for 1% of the total sun UV radiation; and
➢ UV radiation C with a wavelength of 100–280 nanometers which is completely absorbed by the atmosphere and the ozone layer [10].

Bitumen modifications with nanomaterials and polymers have increasingly been studied in recent years to enhance the chemical and mechanical performance characteristics of bitumen. The chemical composition of nanoparticles (NPs), their shape, their ratio of surface area to volume, and the degree of activated bonding at the aggregate–bitumen interface are among the most important parameters studied [11,12,13,14]. 

Some researchers investigated different nanomaterials in their capacity to improve bitumen aging resistance to UV radiation, such as titanium dioxide [15], graphene oxide [16], montmorillonite [17], and zinc oxide [7] (see Table 1). As for titanium dioxide (TiO_2_) and zinc oxide (ZnO), it was found that a photocatalytic process under sunlight may destroy the structure of binder composites with these NPs [18,19,20,21]. Triazine derivatives, benzotriazoles, benzophenones, and salicylates are used as organic UV absorbers, nevertheless, their thermal stability due to low molecular weight is weak [22,23,24]. However, silica NPs have some advantages such as nontoxic, non-photocatalytic, environmentally friendly, and inorganic UV shielding [25,26,27,28].

Recently, among silica NP families, bitumen modification by means of fumed silica has been studied to improve mechanical and rheological properties as well as UV resistance [29]. Fumed silica is a nonporous and highly dispersed amorphous silicon dioxide which is made by silicon tetrachloride (hydrolysis flame) in an oxyhydrogen gas flame at high temperature. Fumed silica is also known as fumed silica NPs, because it fundamentally consists of nanoparticles [30,31]. The large surface area of 20–500 m^2^/g is a unique character of silica NPs, which causes a distinct interaction among particles resulting in a significant effect on the rheological properties of materials [32,33].

The UV aging of asphalt materials is not a new concept, however, due to complex mechanisms caused by UV aging at a molecular bitumen scale, is also depends on the bitumen type, no unique standard for UV aging exists today. 

The present investigation focuses on the effects of bitumen modification using fumed silica NPs in various contents. The aim of this study was to analyze the effects of fumed silica NPs on the mechanical and chemical properties of aged bitumen. The characteristics of modified samples were studied by the FT-IR and rheological testing techniques.

## 2. Experimental

### 2.1. Materials and Methodology

Fumed silica NPs (Aerosil A200, Evonik, Essen, Germany) and 50/70 penetration grade bitumen (Total Co., Paris, France) were used. The silica particles were specified by a mean particle diameter of 20–50 nm, a pH of 3.7–4.5, a specific surface area of 175–225 m^2^ g^–1^, and SiO_2_ purity of 99.8 wt.-%. The mechanical properties of bitumen were measured by a ductilometer machine 1500 mm digital based on ASTM D113 [41] standard (Infratest, 20–2356, Brackenheim, Germany), a Petrotest^©^ machine for identifying the softening point ring and ball according to ASTM D36 [42] standard (PK A5, Dahlewitz, Germany), and an Anton Paar automatic penetrometer (PNR 12, Dahlewitz, Germany) according to the ASTM D5 [43] standard to determine the needle penetration. Table 2 summarizes the physical bitumen properties determined in this study.

**Figure 1:**
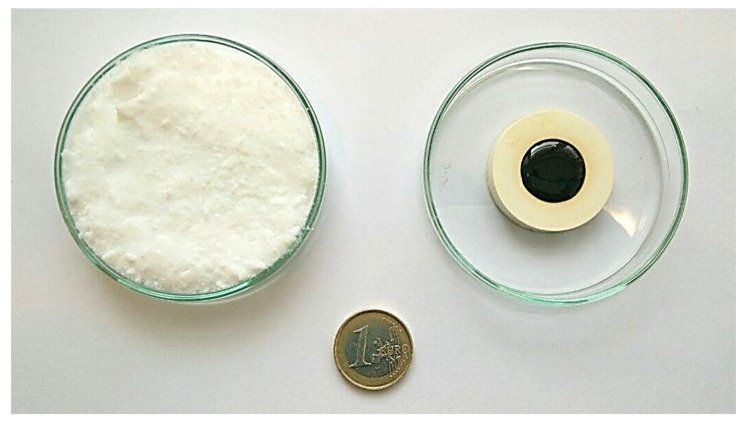
(**left**) Fumed silica nanoparticles and (**right**) the bitumen sample.

Bitumen samples with different concentrations of fumed silica NPs were prepared by mix-melting method. Silica NPs were first dried (to eliminate surface moisture) in an oven at a temperature of 110 °C for 3 h. Then, the bitumen was heated to 150 °C and different contents of NPs (0.1, 2 and 3 wt.-%) were added slowly (to prevent agglomeration). In all steps, temperature was controlled and regularly monitored (Figure 1).

### 2.2. Aging Process

As regards the rolling thin film oven test (RTFOT), the samples of modified bitumen were kept at 163 °C in the rolling thin film oven (RTFOT 8, model of ISL, France) according to the ASTM D1754 standard. The samples were filled in shells of 90 ± 0.5 mm in diameter, and then put in the ultraviolet oven with a UV lamp of 500 W, with an average intensity of 10 W/m^2^ (for 6 and 12 days at 50 °C). In order to prevent thermal oxidation aging as much as possible, the UV process was run at a temperature less than 50 °C. Samples were prepared in three different conditions: unaged samples (S1–S4), samples aged under RTFO conditions (S5–S8), and samples aged under UV conditions (S9–S16). All samples are presented in Table 3.

### 2.3. Characterization Methods

**Figure 2:**
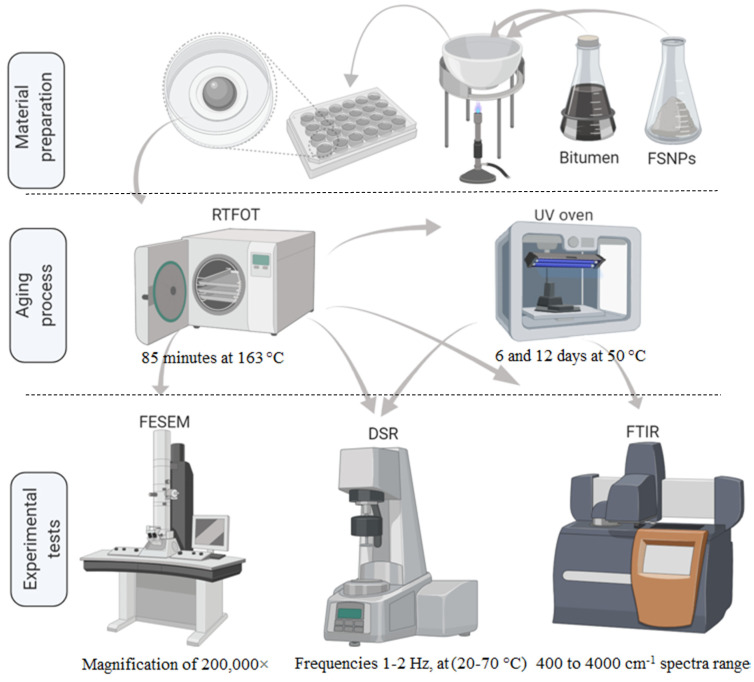
Schematic diagram indicating the material preparation, laboratory aging process, and experimental tests.

In this study, dynamic shear rheometer (DSR) tests were performed to analyze the rheological properties of the modified bitumen samples. Fourier transform infrared spectroscopy (FT-IR) and field emission scanning electron microscopy (FESEM) were used for investigating the chemical structure and morphological properties, respectively. Schematic information on these test methods is reported in Figure 2.

#### 2.3.1. Fourier Transform Infrared Spectroscopy Tests (FT-IR)

Samples were tested through FT-IR in transmission mode between 400 AND 4000 cm^−1^ spectra range (Nexus, Thermo Nicolet Corp., Franklin, MA, USA) at 25 °C. The chemical structure of materials was determined with a range of spectra in different chemical bands. The bitumen samples (0.1 gr) were dissolved in carbon disulfide (of 2 wt.-%), put on a blank Potassium Bromide table and analyzed with FT-IR. Carbonyl and sulfoxide were used as an index for oxidation and short-term aging. Sulfoxide and carbonyl groups were determined and compared in the 1700 and 1030 cm^−1^ wave numbers range [34].

#### 2.3.2. Rheological Tests

A dynamic shear rheometer (DSR, Kinexus DSR, Malvern Panalytical, Malvern, UK) was used to evaluate the bitumen rheological properties in the domain of linear viscoelastic behavior, under different conditions (frequencies 1–2 Hz, and temperatures of 20–70 °C). The bitumen samples were investigated between the parallel plates with a 1 mm gap and 25 mm plate diameter. The complex shear modulus (G*), phase angle (δ), and rutting factor (G*/sinδ) of the control bitumen (unaged) and aged bitumen samples were investigated based on AASHTO T315 standard.

#### 2.3.3. Field Emission Scanning Electron Microscope Tests (FESEM)

A high-resolution FESEM (TE-SCAN, MIRA III, Brno, Czech Republic) was used to consider and validate the micro- and nanostructures of the additives within the bitumen samples. The morphology was characterized by focusing an electron beam on the sample surface with a magnification of 200,000× and a working distance of 4.6 mm.

## 3. Results and Discussion

### 3.1. Surface Morphology

**Figure 3:**
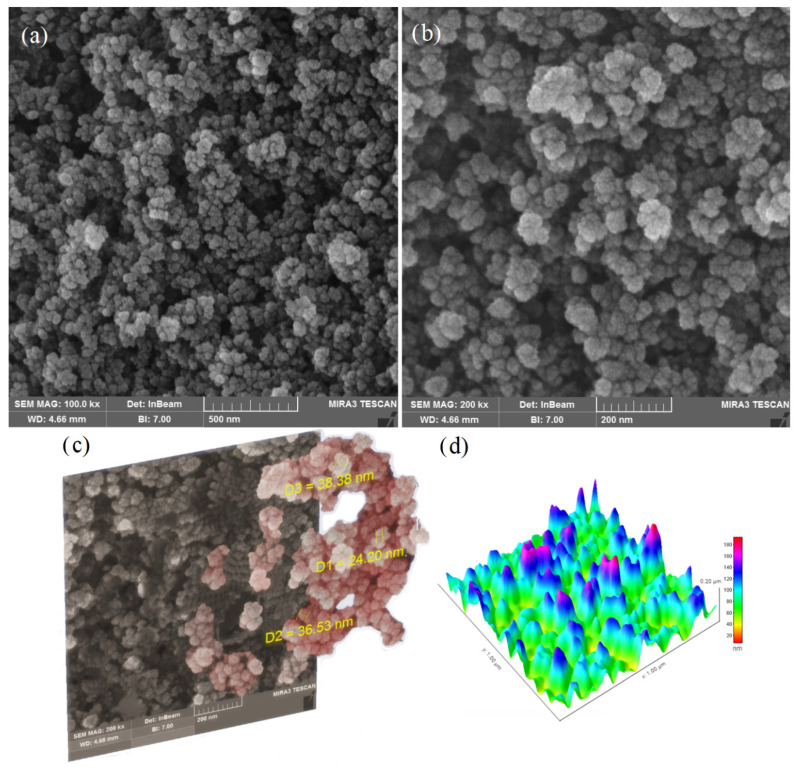
Image analysis of bitumen modified with fumed silica NPs: (**a**,**b**) FESEM; (**c**) FESEM process; and (**d**) surface morphology.

The dispersion of fumed silica NPs in the bitumen samples was evaluated by FESEM observations. Therefore, as shown in FESEM images (Figure 3), uniformly dispersed particles of fumed silica NPs were observed in the bitumen samples, which indicates that fumed silica NPs are separated by melt blending.

FESEM images were analyzed by TESCAN MIRA software to measure the scale of layers and the diameter of particles. Furthermore, the images were then processed with ImageJ, an open source software for digital analyses. Figure 3 shows a complex network structure of fumed silica NPs of wasp nest shape (hexagonal cells). This nanostructure absorbs and reflects UV light, and like a UV shield, it prevents the destruction of the upper structure. At the same time, it traps volatile compounds, and decelerates their loss from bitumen [44]. Considering the large surface of fumed silica NPs, they perfectly coat the bitumen molecules, and while forming a complex network, they cover a wide area. Therefore, a suitable dispersion of the fumed silica NPs in the bitumen is the most important. The average particle size of fumed silica NPs is about 33 nm, as depicted in Figure 3c. The fumed silica NPs form a distinct 3-dimensional pattern, as highlighted in Figure 3d. The arrangement of silica NPs on the bitumen surface was analyzed by topographic imaging technique. From the spectrum of topographic images, it can be seen that the aggregation of fumed silica NPs increased in the violet zones. In fact, whatever peaks higher, aggregation is increased and thus the optimal surface is decreased.

**Figure 4:**
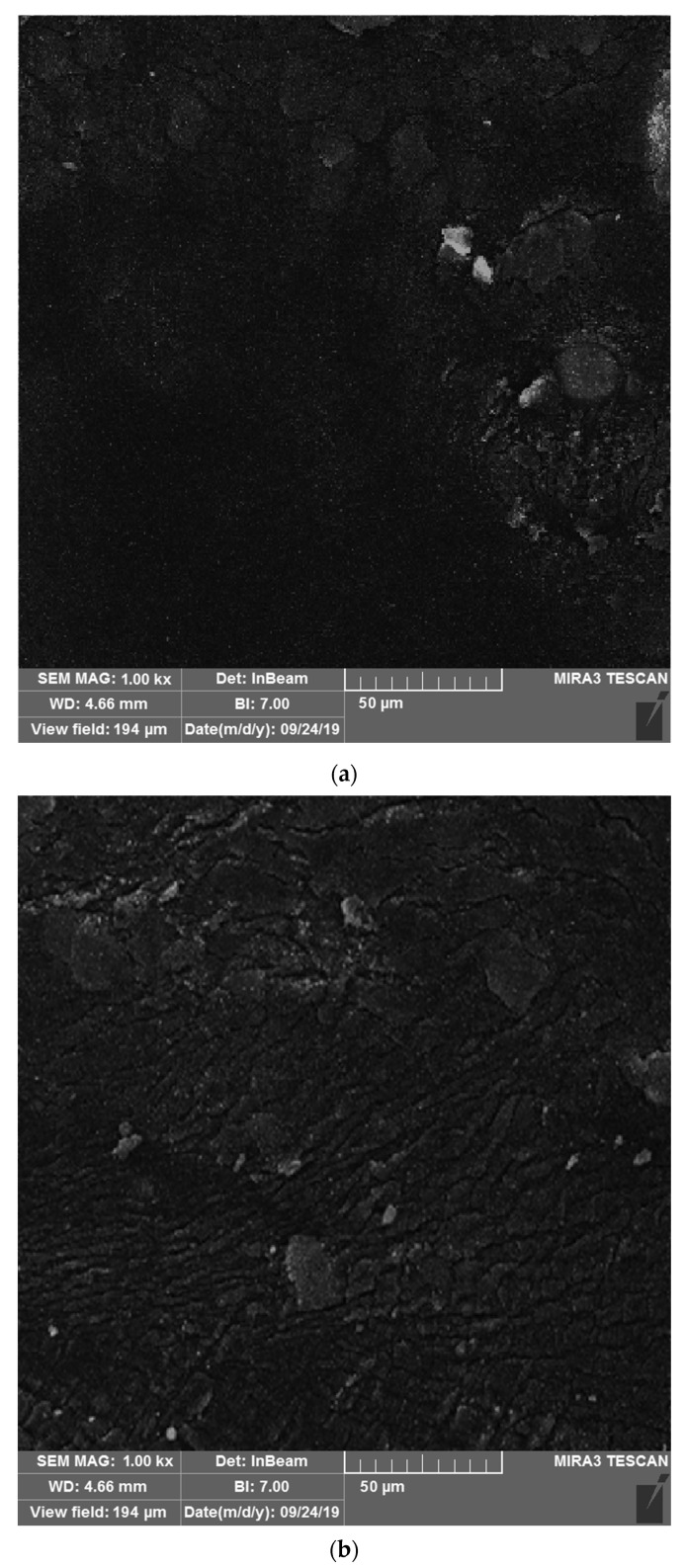
FESEM images of the bitumen modified with fumed silica NPs: (**a**) unaged and (**b**) aged in laboratory.

Figure 4a,b illustrate the effect of fumed silica NPs on the microstructure of bitumen. A change in the surface microstructure is observed, when comparing the surface images of the unaged bitumen (modified with fumed silica NPs) and those of the aged bitumen. It is assumed that the topography change in bitumen surface is related to the release of molecular groups as a consequence of aging [45], and that a more homogeneous structure will protect the bitumen better from aging.

### 3.2. FT-IR Analysis

Carbonyl and sulfoxide bonds are created by the UV radiation process from carbon-carbon or carbon–hydrogen bonds. Therefore, carbonyl (C=O) and sulfoxide (S=O) functions were monitored with spectra of 1700–1030 cm^−1^, respectively. These parameters were selected to indicate the range of oxidation. The C=O group index (I_C=O_), S=O group index (I_S=O_), and change rate (CR) were calculated by Equations (1)–(3) [34,46]:

(1) $$I_{C=O}=\frac{\mathrm{Area}\;\mathrm{of}\;\mathrm{carbonyl}\;\mathrm{band}\;\mathrm{centered}\;\mathrm{around}\;1700{\;\mathrm{cm}}^{-1}}{\sum^{\;}\mathrm{Area}\;\mathrm{of}\;\mathrm{spectral}\;\mathrm{bands}\;\mathrm{around}\;1460\;\mathrm{and}\;1375{\;\mathrm{cm}}^{-1}}$$

(2) $$\;I_{S=O}=\frac{\mathrm{Area}\;\mathrm{of}\;\mathrm{carbonyl}\;\mathrm{band}\;\mathrm{centered}\;\mathrm{around}\;1030{\;\mathrm{cm}}^{-1}}{\sum^{\;}\mathrm{Area}\;\mathrm{of}\;\mathrm{spectral}\;\mathrm{bands}\;\mathrm{around}\;1460\;\mathrm{and}\;1375{\;\mathrm{cm}}^{-1}}$$

(3) $$\mathrm{CR}=\frac{\mathrm{Index}\;\mathrm{of}\;\mathrm{bitumen}\;\mathrm{after}\;-\mathrm{Index}\;\mathrm{of}\;\mathrm{bitumen}\;\mathrm{before}}{\mathrm{Index}\;\mathrm{of}\;\mathrm{bitumen}\;\mathrm{before}}$$

**Figure 5:**
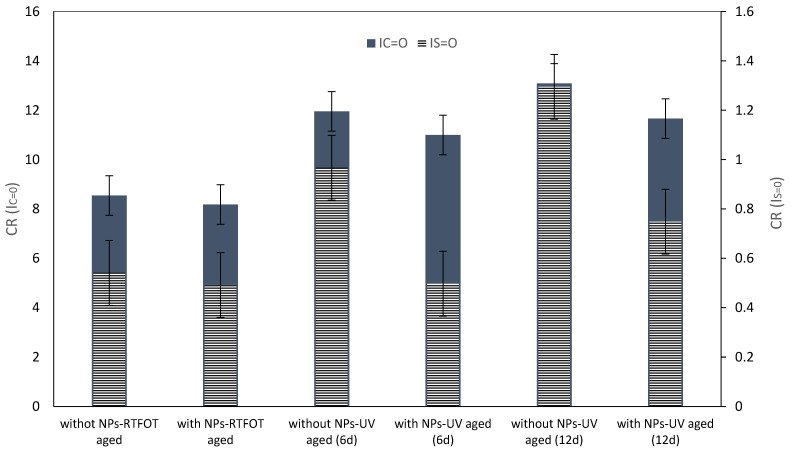
Structural index of bitumen before and after laboratory aging.

The results are reported in Figure 5. For UV aged samples, the carbonyl index increased more than for the control bitumen. Furthermore, carbonyl index and sulfoxide index increased when the aging time increased. The index of carbonyl increased to 0.0063, 0.0086, and the sulfoxide index increased to 0.037 and 0.064 after 6 and 12 days, respectively, which was related to the duration of UV radiation.

In opposition to the bitumen modified with fumed silica NPs, both the carbonyl index and the sulfoxide index decreased. Obviously, the added fumed silica NPs created an efficient UV-shielding coating [47].

### 3.3. Viscoelastic Properties

#### 3.3.1. Complex Modulus and Phase Angle

**Figure 6:**
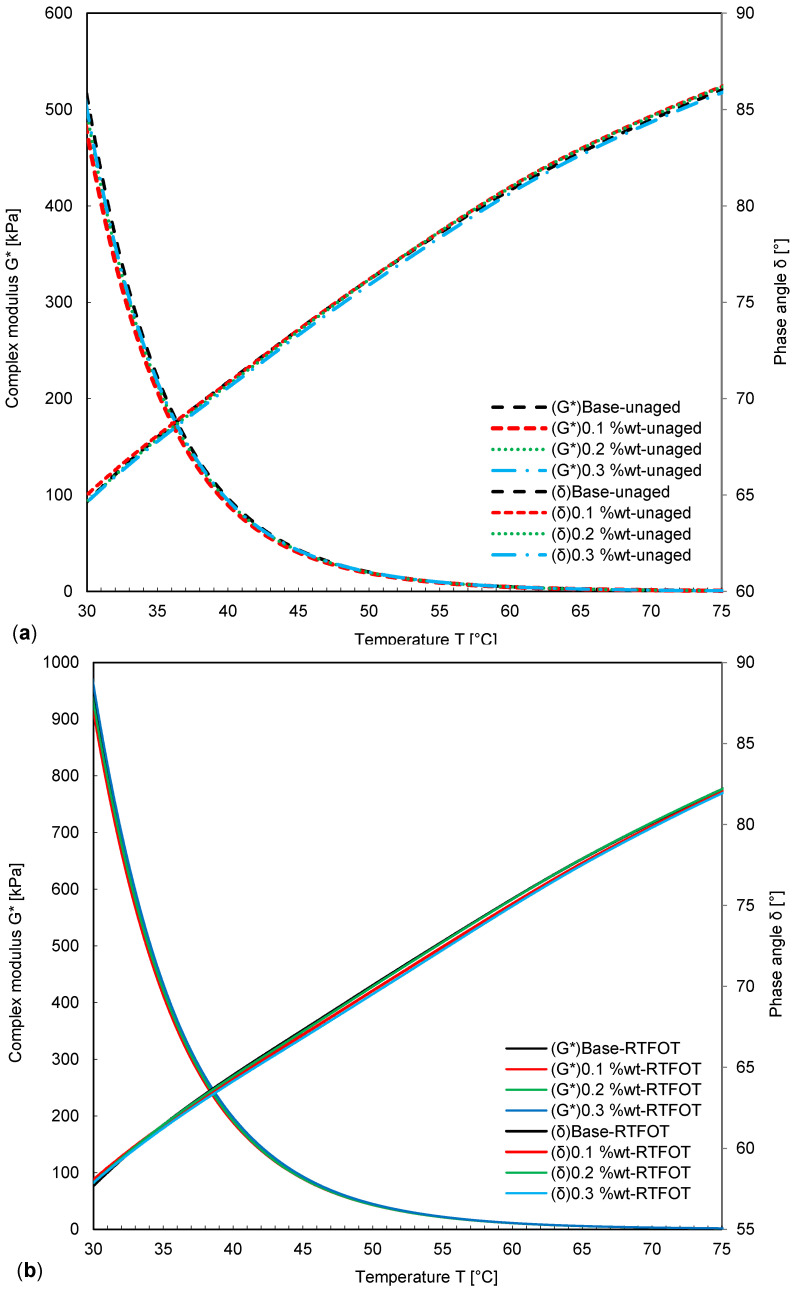
Complex modulus and phase angle of bitumen modified with NPs: (**a**) before and (**b**) after short-term laboratory aging.

**Figure 7:**
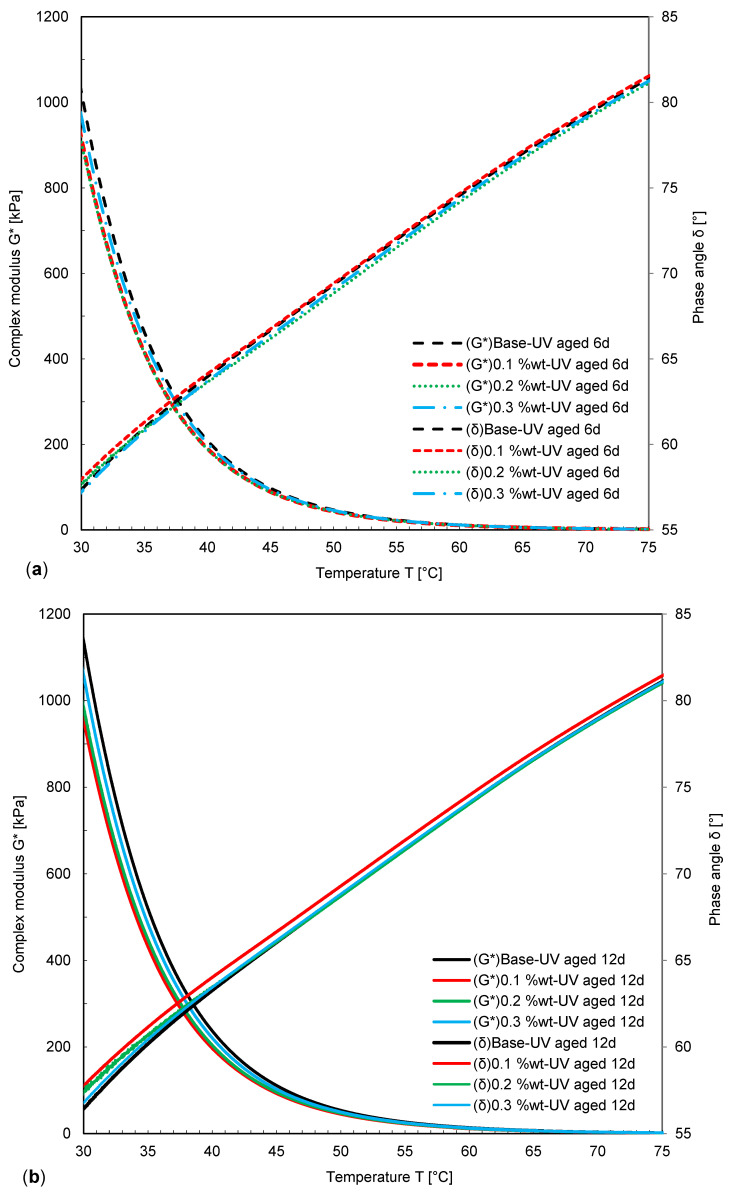
Complex modulus and phase angle of modified bitumen with NPs after (**a**) 6 days of UV aging and (**b**) after 12 days of UV aging.

The results of DSR tests in terms of the complex modulus and phase angle from 20 to 70 °C are presented in Figure 6 and Figure 7. Before aging, the level of complex modulus was higher for samples modified with NPs, resulting in the best deformation resistance for sample S4. Complex modulus ranking of samples before aging is with 0.1 wt.-% NPs < with 0.2 wt.-% NPs < without NPs < with 0.3 wt.-% NPs; and the ranking of the phase angle is: with 0.1 wt.-% NPs > with 0.2 wt.-% NPs > without NPs > with 0.3 wt.-% NPs.

Obviously, the UV aging results in increased stiffness and therefore, the complex modulus of samples was increased. Hence, the maximum complex modulus was observed for the Sample with 0.3 wt.-% NPs. In contrast to the complex modulus, the value of the phase angle was always decreased as a consequence of aging.

The change in complex modulus also depends on the amount of fumed silica NPs added to the bitumen. After short-term aging and UV aging, the ranking of the complex modulus and the phase angle were found as: 0.1 wt.-% NPs < 0.2 wt.-% NPs < without NPs < 0.3 wt.-% NPs, and 0.1 wt.-% NPs < 0.2 wt.-% NPs < 0.3 wt.-% NPs < without NPs.

UV aging results illustrated that increasing the duration of UV aging significantly affected the complex modulus, while NPs subdued this effect, which was the most distinct for a NP concentration of 0.1 wt.-%. Hence, the optimum range of NP concentration in bitumen was assumed to be between 0.1 and 0.2 wt.-%, as for the sample with 0.1 wt.-% NPs, the minimum phase angle and the maximum complex modulus were obtained. However, considering short-term aging, fumed silica NPs showed only a low effect on the phase angle and complex modulus. It was concluded, that 0.1 wt.-% of fumed silica NPs can be successfully used as a UV shield. Moreover, the effect of fumed silica NPs on UV aging was more distinct after 6 and 12 days due to their shielding and absorption effect, rather than short-term aging.

#### 3.3.2. Resistance to Permanent Deformation

**Figure 8:**
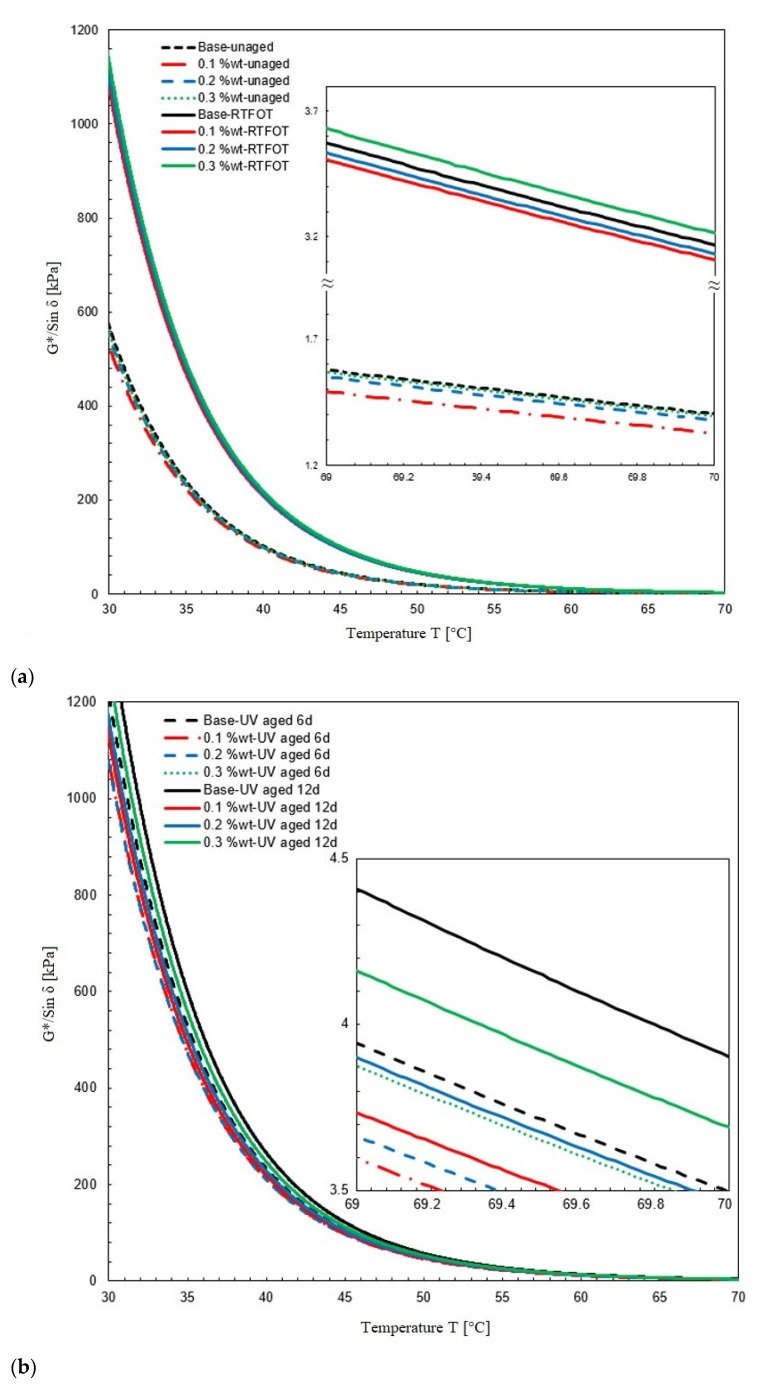
Resistance to the permanent deformation of bitumen samples after (**a**) short-term aging, and (**b**) UV aging.

Figures indicating the resistance to permanent deformation (rutting factors) between temperatures of 20 and 70 °C before and after aging are displayed in Figure 8. Obviously, NPs improved the bitumen’s resistance to permanent deformation. In Figure 8b, the values for S10 are the smallest after aging, which shows that a certain concentration of fumed silica NPs lead to a good compatibility against destruction due to UV radiation.

The increase in rutting resistance can be analyzed by considering the threshold temperatures of the rutting factor [48,49]. Table 4 summarizes the temperatures found at a rutting factor of 1 before aging, and of 2.2 kPa after aging, respectively (according to the SHRP-A-369 standard). These results showed that NPs decreased the bitumen rutting resistance before aging. After aging, the threshold temperature decreased with the addition of fumed silica NPs. The threshold temperature of samples after aging were found as 72.8, 73.1 and 73.4 for S6, S10, and S14, respectively. These values confirm that fumed silica NPs additives reduce the stiffness level of bitumen samples in UV aging, and moreover, the results showed that 0.1 wt.-% of fumed silica NPs go together with a better performance after UV aging than higher concentrations.

### 3.4. Mechanical Properties

The validation of FT-IR results with rheological aging properties, such as the index of viscosity aging and index of complex modulus aging, is usually used for differently modified bitumen [48]. In this study, the index of viscosity aging (IVA) was used to investigate the aging properties:

IVA = (viscosity of bitumen after aging − viscosity of unaged bitumen)/viscosity of unaged bitumen(4)

**Figure 9:**
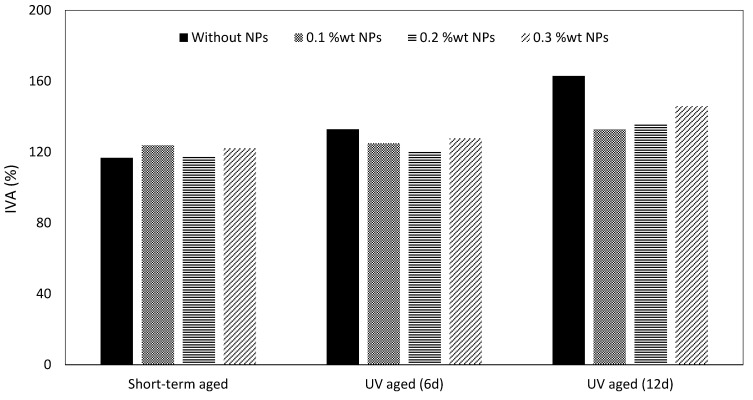
Index of viscosity aging (IVA) of the control and modified bitumen.

After short-term and UV aging, the index of viscosity aging for the samples modified with NPs was found to be smaller than that for control samples, which confirms the increase in bitumen aging resistance with fumed silica NPs (Figure 9). The results also indicate that increasing the duration of UV aging causes an increase in the index of viscosity aging. Samples with 0.2 wt.-% and 0.1 wt.-% NPs resulted in the smallest level of IVA after 6 and 12 days, respectively. Moreover, increasing the fumed silica NP concentration in bitumen resulted in an increase in IVA and in a reduction in the aging resistance. This phenomenon is related to the Derjaguin–Landau–Verwey–Overbeek (DLVO) theory, dispersion stability, and the optimum aggregation concentration range which can be very important in the preparation of asphalt mixture and polymer-modified bitumen [50,51,52,53,54].

## 4. Conclusions

In this experimental study, the effects of UV aging and the short-term aging of bitumen samples were studied, considering various concentrations of fumed silica NPs in the bitumen. The performance of the bitumen samples in terms of rheological and chemical properties was analyzed. Based on this investigation, the main outcomes were as follows:

The FT-IR results showed that the addition of fumed silica NPs to bitumen improved the aging resistance of bitumen, because of the UV shielding effects of the NPs. After UV aging, the carbonyl and sulfoxide index decreased in bitumen samples modified with NPs. Bitumen samples were tested, having three different concentrations of fumed silica NPs. According to the specific concentration of NPs, the UV and short-term aging resistance of bitumen changed significantly. Low concentrations of NPs reduce the stiffness level, and a concentration of 0.1 wt.-% was found to result in the best performance.

Rheological tests confirmed that increasing the duration of UV aging significantly affected the complex modulus, while adding fumed silica NPs to bitumen considerably improved the bitumen aging resistance, also recorded by a significant reduction in the index of viscosity aging. The effect of fumed silica NPs on UV aging was more distinct due to their shielding and absorption effect than short-term aging. In addition, the rutting factor results show that NPs improved the bitumen’s resistance to permanent deformation.

## Figures and Tables

**Table 1:** Effects of UV aging on nanomaterial additives, as observed by various researchers.

Nanomaterial Additives	Content	Intensity of UV Radiation	Bitumen Thickness	Aging Time	Aging Temperature (°C)	Observed Effect	References
Montmorillonite	5 wt.-%	2800 μW/cm^2^	-	288 h	60	Used two types of montmorillonite (PMMT and OMMT)/improved UV resistance	[34]
Titanium dioxide/montmorillonite	4–6 wt.-%	3.18 W/cm^2^	1 mm	336 h	-	5 wt.-% of the modifier provided was the best UV aging resistance for the bitumen	[15]
Zinc oxide	2–3 wt.-%	-	-	6 d	80	3 wt.-% has the best anti-aging performance	[35]
Mg–Al–CO_3_ layered double hydroxides	3 wt.-%	1.2 W/cm^2^	-	9 d	60	Layered double hydroxides with 180 nm has the strongest ability to absorb and reflect UV light	[36]
Graphene oxide and carbon nanotubes	1–3 wt.-%	129 W/m^2^	1.5 mm	12 d	45	3 wt.-% was better than that of 1 wt.-% GO/improved the UV aging resistance	[37]
Clay	1–3 wt.-%	10 W/m^2^	1 mm	12 d	60	2% had a better performance/improved the UV aging resistance	[17]
Titanium dioxide	3 wt.-%	27.58 W/cm^2^	-	43 d	60	0.3 wt.-% TiO_2_ + 0.1 wt.-% butylated hydroxytoluenecan significantly reduced the UV aging rate	[38]
Graphene oxide	0.5–1.5 wt.-%	2000 μW/cm^2^	3.2	9 d	50	1.5 wt.-% has the best anti-aging performance	[16]
Zinc oxide	1–5 wt.-%	-	3.2 mm	400 h	-	The reasonable dosage of nano-ZnO for anti-aging performance determined as 3.0 wt.-%	[39]
Titanium dioxide	1–5 wt.-%	8 W/m^2^	-	6 d	60	improve the UV aging resistance	[40]

**Table 2:** Specification of the penetration grade bitumen 50/70.

Physical Properties	Ductility (@ 25 °C, cm)	Softening Point (°C)	Penetration (@ 25 °C, 0.1 mm)	Density (kg/m^3^)
Value	100	48.6	63	1.03
Standard	ASTM D113	ASTM D36	ASTM D5	ASTM D70

**Table 3:** Samples with different additives, and conditioning according to different aging processes (unaged, RTFO aged, and UV aged).

Sample No.	NPs Additives	Aging Process	Sample No.	Additives	Aging Process
S1	-	Unaged	S9	-	6 d UV
S2	0.1 % wt NPs	Unaged	S10	0.1 % wt NPs	6 d UV
S3	0.2 % wt NPs	Unaged	S11	0.2 % wt NPs	6 d UV
S4	0.3 % wt NPs	Unaged	S12	0.3 % wt NPs	6 d UV
S5	-	RTFO	S13	-	12 d UV
S6	0.1 % wt NPs	RTFO	S14	0.1 % wt NPs	12 d UV
S7	0.2 % wt NPs	RTFO	S15	0.2 % wt NPs	12 d UV
S8	0.3 % wt NPs	RTFO	S16	0.3 % wt NPs	12 d UV

**Table 4:** Bitumen threshold temperatures under different concentrations of fumed silica NPs before and after aging.

Threshold Temperatures (°C)															
Before Aging (G*/sin δ = 1 kPa)				After Aging (G*/sin δ = 2.2 kPa)											
S1	S2	S3	S4	S5	S6	S7	S8	S9	S10	S11	S12	S13	S14	S15	S16
72.8	72.3	72.7	72.8	73.0	72.8	72.9	73.1	73.9	73.1	73.8	73.2	74.8	73.4	74.3	73.8

## Data Availability

Not applicable.

## Abbreviation

DSR	Dynamic shear rheometer
wt.-%	weight-%
FT-IR	Fourier transform infrared spectroscopy
SEM	Scanning electron microscopy
UV	Ultraviolet
RTFOT	Rolling thin film oven test
NPs	Nanoparticles
IVA	Index of viscosity aging
I_C=O_	C=O group index
I_S=O_	S=O group index
CR	Change rate
DLVO	Derjaguin–Landau–Verwey–Overbeek
